# Natural compounds targeting glycolysis as promising therapeutics for gastric cancer: A review

**DOI:** 10.3389/fphar.2022.1004383

**Published:** 2022-11-10

**Authors:** Maoyuan Zhao, Feng Wei, Guangwei Sun, Yueqiang Wen, Juyi Xiang, Fangting Su, Lu Zhan, Qing Nian, Yu Chen, Jinhao Zeng

**Affiliations:** ^1^ Department of Oncology, Hospital of Chengdu University of Traditional Chinese Medicine, Chengdu, China; ^2^ Department of Oncology, Sichuan Integrative Medicine Hospital, Chengdu, China; ^3^ School of Basic Medicine, Chengdu University of Traditional Chinese Medicine, Chengdu, China; ^4^ TCM Regulating Metabolic Diseases Key Laboratory of Sichuan Province, Hospital of Chengdu University of Traditional Chinese Medicine, Chengdu, China; ^5^ Department of Blood Transfusion, Sichuan Provincial People’s Hospital, University of Electronic Science and Technology of China, Chengdu, China; ^6^ Geriatric Department, Hospital of Chengdu University of Traditional Chinese Medicine, Chengdu, China

**Keywords:** gastric neoplasm, Warburg effect, glycolysis, phytochemicals, biological products

## Abstract

Gastric cancer, a common malignant disease, seriously endangers human health and life. The high mortality rate due to gastric cancer can be attributed to a lack of effective therapeutic drugs. Cancer cells utilize the glycolytic pathway to produce energy even under aerobic conditions, commonly referred to as the Warburg effect, which is a characteristic of gastric cancer. The identification of new targets based on the glycolytic pathway for the treatment of gastric cancer is a viable option, and accumulating evidence has shown that phytochemicals have extensive anti-glycolytic properties. We reviewed the effects and mechanisms of action of phytochemicals on aerobic glycolysis in gastric cancer cells. Phytochemicals can effectively inhibit aerobic glycolysis in gastric cancer cells, suppress cell proliferation and migration, and promote apoptosis, *via* the PI3K/Akt, c-Myc, p53, and other signaling pathways. These pathways affect the expressions of HIF-1α, HK2, LDH, and other glycolysis-related proteins. This review further assesses the potential of using plant-derived compounds for the treatment of gastric cancer and sheds insight into the development of new drugs.

## 1 Introduction

Cancer is the second leading cause of death among humans. In 2020, approximately 19.3 million new cases and 10 million deaths were reported worldwide due to cancer (Sung et al., 2021). Gastric cancer has the fifth-highest rate of global incidence among cancers and is ranked fourth in mortality rate (Sung et al., 2021). *Helicobacter pylori* (*Hp*) infection is considered to be the main cause of gastric cancer, and administering the *Hp* eradication therapy promptly can effectively reduce the incidence of gastric cancer (Hooi et al., 2017; Liou et al., 2020). Heredity, smoking, consumption of alcohol, and improper diet increase the risk of gastric cancer (Rawla and Barsouk, 2019). Endoscopic surgery and gastrectomy are the treatment strategies adopted for early-stage gastric cancer, while adjuvant or neoadjuvant chemotherapy can be used for treatment of locally advanced gastric cancer, as these increase the possibility of surgical resection (Evans et al., 2015). Palliative chemotherapy and molecular targeted therapy are commonly administered to patients with unresectable, locally advanced, and metastatic tumors (Shen et al., 2013; Waddell et al., 2013; Ajani et al., 2016). However, chemotherapy results in severe systemic toxicity, including peripheral neuropathy, nausea and vomiting, mucosal inflammation, and suppression of bone marrow functions (Gibson and Keefe, 2006). The development of resistance to drugs is also the main cause of the failure of chemotherapy and targeted therapy (Roviello et al., 2022; Zhang et al., 2022). Taken together, these reasons have resulted in a high mortality rate of gastric cancer.

At present, molecular targeted therapy is an important avenue in cancer research, including the discovery of molecular targets specifically expressed by tumor cells and the development of corresponding drugs that can accurately inhibit tumor cell activity (Yim and Cunningham, 2011). In the 1920s, Otto Warburg discovered that tumor cells produced energy through the glycolytic pathway even under aerobic conditions (Liberti and Locasale, 2016). This is known as the Warburg effect, or aerobic glycolysis, and is a common feature observed in rapidly proliferating cells. The Warburg effect facilitates cancer cells to rapidly transport and consume glucose, and generate ATP to meet their energy needs. Aerobic glycolysis simultaneously produces several macromolecular compounds that can be used for lipid and protein synthesis, to meet the requirements of growing cells. A large amount of lactic acid produced by aerobic glycolysis can be transported by the monocarboxylate transporters (MCTs) to the extracellular space, resulting in an extracellular acidic environment. This environment is conducive to the immune escape of tumor cells and promotes their proliferation, invasion, and metastasis (Vander Heiden et al., 2009; Upadhyay et al., 2013). Owing to the Warburg effect, the targeted killing of gastric cancer cells by the inhibition of aerobic glycolysis is feasible (Chen et al., 2007).

The occurrence of gastric cancer is related to the diet of an individual; numerous studies have confirmed that the long-term consumption of certain fruits and vegetables can prevent the occurrence of gastric cancer (Steevens et al., 2011). *In vitro* experiments have also confirmed that phytochemicals can inhibit proliferation and metastasis, induce apoptosis and autophagy, and inhibit the angiogenesis of gastric cancer cells (Mao et al., 2020). Many phytochemicals have been used to treat tumors (Cheng et al., 2021). For example, paclitaxel, extracted from the bark of *Taxus brevifolia*, is widely used in the clinical treatment of cancers of the breast, lungs, and ovaries. Camptothecin analogs, mainly camptothecin and irinotecan, extracted from *Camptotheca acuminate*, are used in the treatment of multiple solid tumor types (Mann, 2002). Aneustat (OMN54), a mixture of extracts of *Ganoderma lucidum*, *Salvia miltiorrhiza* and *Scutellaria barbata*, has shown favorable anticancer effect in a Phase-I Clinical Trial (NCTId: NCT01555242) (Renouf et al., 2015). *In vitro* experiments showed that Aneustat effectively inhibited the aerobic glycolysis of LNCaP prostate cancer cells (Qu et al., 2018). Silybin (SIL) is a GLUT inhibitor, and its main component is flavonoids extract from *Silybum marianum*. Phase I trials have confirmed its therapeutic effect on prostate cancer (Flaig et al., 2007). Other aerobic glycolysis inhibitors such as AZD3965 (MCT1 inhibitor) (Szliszka et al., 2009), 2-deoxyglucose (2-DG, G6PI inhibitor) (Dwarakanath et al., 2009), CPI -613 (PDH inhibitor) (Senzer et al., 2012), AZD3965 (MCT1 inhibitor) (Kennedy and Dewhirst, 2010), have achieved exciting results *in vitro* and *in vivo* experiments and have entered the clinical trial stages or been applied clinically. Therefore, the identification of new drugs from plant compounds that target gastric cancer cells is necessitated. In this review, we discuss promising pharmacological strategies for the use of phytochemicals with anti-glycolytic activities for the treatment of gastric cancer.

## 2 Role of aerobic glycolysis in gastric cancer

Active aerobic glycolysis in tumor cells is associated with several substances. Glucose transporters (GLUTs) present on cell membranes mediate the entry of glucose into the cytoplasm through diffusion and are overexpressed in cancer cells, especially GLUT1. Following the absorptions of glucose, hexokinase (HK) catalyzes the process of glucose phosphorylation to produce glucose 6-phosphate (G6P), which is then catalyzed by phosphofructokinase-1 (PFK-1), pyruvate kinase (PK), lactate dehydrogenase (LDH), and other enzymes, to generate energy and various metabolic substrates that can be used for the synthesis of lipids, amino acids, and other substances in tumor cells (Hatzivassiliou et al., 2005; Vander Heiden et al., 2009; Lunt and Vander Heiden, 2011; Huang et al., 2014). As the end product of glycolysis, pyruvic acid can be transported to the mitochondria, wherein it is converted by pyruvate dehydrogenase (PDH) to acetyl-CoA, thus powering the tricarboxylic acid (TCA) cycle (Kim and Dang, 2006; Vander Heiden et al., 2009). Under hypoxic conditions, LDH catalyzes the conversion of pyruvic acid into lactic acid, which can be transported to the extracellular space by MCTs. Extracellular lactic acid enters cells along with adequate oxygen supply, it is converted into pyruvate by PDH, catalyzed into acetyl-CoA, and enters the TCA cycle (Brooks, 2018). The accumulation of lactic acid in the microenvironment reduces T-cell activity and inhibits the cytotoxicity of natural killer cells (Kumar and Gabrilovich, 2014; Huber et al., 2017). It also promotes phenotypic polarization of tumor-associated macrophages to M2, leading to tumor immune escape (Shapouri-Moghaddam et al., 2018; de la Cruz-López et al., 2019). Additionally, the acidic microenvironment is conducive to the up-regulation of matrix metalloproteinases (MMPs), urokinase-type plasminogen activator (u-PA), and cathepsin, resulting in the remodeling of the extracellular matrix and cellular migration (Sloane et al., 1990; Szpaderska and Frankfater, 2001; Kindzelskii et al., 2004; Gatenby et al., 2006; Kato et al., 2007; Smallbone et al., 2007). Lactic acid also promotes tumor cell angiogenesis, facilitating conducive conditions required for tumor cell development, infiltration, and metastasis (Dhup et al., 2012; Polet and Feron, 2013; Icard et al., 2018).

Various signaling pathways are involved in the mediation of aerobic glycolysis in cancer cells. Hypoxia-inducible factor-1 (HIF-1) is a key transcription factor that promotes aerobic glycolysis. It is highly expressed in gastric cancer cells and promotes the expression of various glycolysis-related proteins. HIF-1 is a heterodimer comprising HIF-1α and HIF-1β subunits, of which, the former is hydroxylated by prolyl hydroxylases (PHs) under normoxia, further triggering ubiquitination and proteasomal degradation (Marín-Hernández et al., 2009). The activity of PHs is highly regulated by the intracellular oxygen content and is inhibited under hypoxic conditions, resulting in decreased HIF-1α degradation and increased HIF-1 dimerization (Philip et al., 2013). The tumor suppressor gene, p53, inhibits the transcription of GLUT1 and GLUT4 in a tissue-specific manner and reduces the levels of fructose-2,6-bisphosphate (F-2, 6-BP), thereby inhibiting glycolysis by inducing TP53-induced glycolysis and apoptosis regulator (TIGAR) with TP53 (Schwartzenberg-Bar-Yoseph et al., 2004; Bensaad et al., 2006). The proto-oncogene, c-Myc, up-regulates the expression of several key glycolytic enzymes, which in turn promote glycolysis. c-Myc can bind to the regulatory region of hexokinase 2 (HK2), and directly facilitate the enrichment of the promoter region of pyruvate kinase M2 (PKM2) and up-regulate the expressions of HK2 and PKM2 (Dejure and Eilers, 2017; Gupta et al., 2018). Adenosine monophosphate-activated protein kinase (AMPK) can sense changes in the levels of energy metabolism in the cells and regulates glucose metabolism (Ojuka, 2004). AKT phosphorylation up-regulates the expressions of GLUTs and HK2, improves the activity of glycolysis and mammalian targets of the rapamycin (mTOR) (Courtney et al., 2010), and stimulates glycolysis by inducing the expression of HF1-α, a major signaling pathway involved in the regulation of aerobic glycolysis and tumor growth (Rosen and She, 2006; Xie et al., 2019).

**FIGURE 1:**
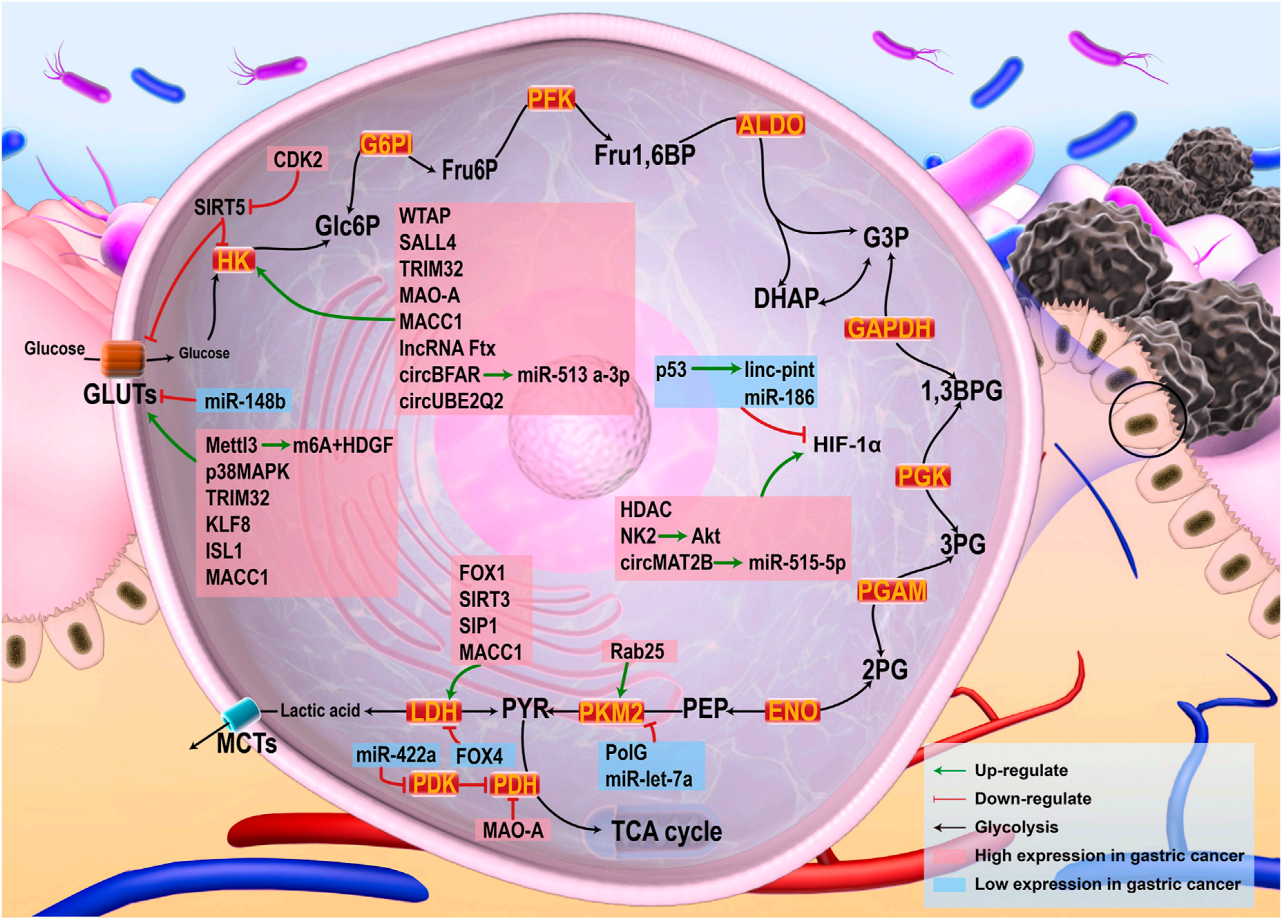
Glycolysis in cancer cells and the related regulatory pathways. Glucose was uptaken into cells by GLUTs, where it is metabolized into pyruvic acid by a series of enzyme reactions. Pyruvic acid could be metabolized by LDH to lactic acid out of the cells, or by PDH to acetyl-CoA into the TCA cycle when oxygen supply is adequate. HIF-1, the main regulator of glycolysis, was able to up-regulate the expression of a variety of enzymes in the glycolysis process. Multiple tumor-related pathways were involved in regulating glycolysis of gastric cancer. Factors with high expression in gastric cancer, such as HDAC and WTAP, can directly promote glycolysis of gastric cancer by up-regulating the expression of glycolysis-related proteins or enzymes. Some factors that can inhibit glycolysis-related enzymes, such as FOX4, miR-186, *etc.*, were low expressed in gastric cancer.

Multiple studies have confirmed that the occurrence and development of gastric cancer are related to active aerobic glycolysis, and various molecular mechanisms have been implicated (Figure 1). *Hp* infection, the most common cause of gastric cancer, leads to elevated levels of reactive oxygen species (ROS) in tumor cells, resulting in the inhibition of PH activity and increased HIF-1 levels, which promote aerobic glycolysis (Bhattacharyya et al., 2010). Glucose uptake in gastric cancer patients was measured in a previous study using F-fluorodeoxyglucose (FDG)-PET and except for signet-ring cell carcinoma, glucose uptake was enhanced in most gastric cancers exhibiting high levels of GLUT-1 (Alakus et al., 2010). Histone deacetylase (HDAC) is associated with cancer development. High expression of HDAC1 in gastric cancer tissues promotes glycolysis due to an increase in the activity of HIF-1α (Jiang et al., 2019). N6-methyladenosine (m6A) is the most common RNA modifier in eukaryotes, and Wilms tumor 1 associated protein (WTAP) is an m6A methyltransferase that can enhance the stability of HK2 mRNA, thus promoting glycolysis in gastric cancer cases (Yu et al., 2021). Mettl3 is involved in the modification of m6A RNA, and its high expression in gastric cancer tissues promotes the mRNA binding of m6A and HDGF (a growth factor). Subsequently, HDGF up-regulates the expression of GLUT4 and other key proteins, thus promoting aerobic glycolysis in gastric cancer cases (Wang Q. et al., 2020). The stem cell factor, SALL4, is activated in gastric cancer cells. It can up-regulate the expression of HK2 and enhance the Warburg effect in gastric cancer cells (Shao et al., 2020). P38MAPK, a member of the MAPK family, shows high expression in gastric cancer cells and promotes glycolysis by up-regulating GLUT4. CDK2, a cell cycle-dependent kinase, is up-regulated in gastric cancer cells (Liu et al., 2015). CDK2 inhibits the expression of SIRT5, which in turn inhibits the expressions of GLUT1, HK2, LDHA, and PDK1 (Tang et al., 2018). Tripartite motif protein 32 (TRIM32), an E3 ubiquitin ligase, promotes glycolysis in gastric cancer cells by targeting GLUT1 and HK2 (Wang J. et al., 2020). The forkhead box (FOX) transcription factor is involved in the regulation of various biological processes. The expression of FOX1 is high in gastric cancer cells and promotes glycolysis by increasing the activity of LDHA (Jiang et al., 2015). FOX4 is down-regulated in gastric cancer cells, and reduces LDHA viability, resulting in enhanced active glycolysis in gastric cancer cells (Wang X. H. et al., 2021). The Krüppel-like transcription factor (KLF) family plays varied roles in several tumorigenesis-related processes. KLF8 expressed at high levels in gastric cancer tissues activates the GLUT4 promoter, and promotes glycolysis in a dose-dependent manner (Mao et al., 2019). Insulin gene enhancer protein 1 (ISL1), a LIM-homeodomain transcription factor, regulates the transcription of GLUT4 and promotes glycolysis in gastric cancer cells (Guo et al., 2021). Sirtuin 3 (SIRT3) can enhance the activity of LDHA and promote glycolysis (Cui et al., 2015). The oncogene, Never in mitosis gene A-related kinase 2 (NK2), promotes glycolysis in gastric cancer cells by activating the Akt/HIF-1α signaling axis (Wan et al., 2021). High levels of Smad interacting protein 1 (SIP1) promote LDHA-mediated glycolysis (Sun et al., 2015). Rab25, a hub node in protein-protein interaction networks, is a positive regulator of PKM2, which promotes glycolysis in gastric cancer cells by increasing the phosphorylation of PKM2 (Zheng et al., 2021). Monoamine oxidase A (MAO-A) is a mitochondrial protein that is up-regulated in gastric cancer cells. It can promote the expression of HK2 and inhibit that of PDH, thus promoting glycolysis in gastric cancer cells (Chen L. et al., 2020). DNA polymerase γ (PolG) is implicated in mitochondrial homeostasis. It can bind to PKM2 and inhibit glycolysis and shows low expression in gastric cancer cells (Lv et al., 2021). Metastasis-associated in colon cancer-1 (MACC1) can promote the Warburg effect by up-regulating the activity and expressions of GLUT1, HK2, LDH, and PDK (Lin et al., 2015a).

Several non-coding RNAs also contribute to aerobic glycolysis in gastric cancer cells. The expression of HK2 is promoted by lncRNA Ftx in gastric cancer cells (Zhu et al., 2020). p53-induced linc-pint promotes the down-regulation of HIF-1α expression in gastric cancer cells (Hong et al., 2019). circMAT2B is a competitive endogenous RNA (ceRNA) that sponges miR-515-5p and increases HIF-1α expression, thereby enhancing glycolysis in gastric cancer cells (Liu J. et al., 2020). CircBFAR promotes glycolysis in gastric cancer cells by targeting the miR-513 a-3p/HK2 axis (Pu et al., 2020). circUBE2Q2 is significantly up-regulated in gastric cancer cells and promotes glycolysis by increasing the levels of HK2 (Yang J. et al., 2021). miR-422a is down-regulated in gastric cancer; it can inhibit PDK2 and restore the activity of PDH, leading to an increased Warburg effect in gastric cancer cells (He et al., 2018). miR-148b, miR-let-7a, and miR-186 inhibit GLUT1 (Ding et al., 2017), PKM2 (Tang et al., 2016), and HIF-1α (Liu et al., 2016), respectively. Low expressions of these miRNAs promote the Warburg effect in gastric cancer cells. Gastric cancer cells exhibit significant characteristics of aerobic glycolysis, and targeting it is a promising strategy for the treatment of gastric cancer.

## 3 Targeting glycolysis in gastric cancer therapy

The high mortality levels associated with gastric cancer can be attributed to the difficulty of early-stage disease diagnosis and the lack of effective therapeutic drugs for the middle-late stages. Active aerobic glycolysis occurs in gastric cancer cells, which can be used to develop related technologies, identify new biomarkers to improve diagnostic efficiency in the early stages or develop drugs targeting glycolysis to improve the survival rate of patients at advanced stages. The glucose analog, 18F-2-fluoro-2-deoxy-D-glucose (18F-FDG), is often used in positron emission computed tomography (PET/CT) scanning. It can display the level of glucose metabolism in organs with high specificity for the detection of distant lymph node metastasis and postoperative recurrence of gastric cancer. However, it has little significance for the classification of gastric cancers exhibiting a low GLUT-1 expression (Lee et al., 2012). Metabonomics aims to analyze the relationship between metabolites and diseases by assessing the endogenous metabolites in biological samples. In a study, the differences in the metabolomics of plasma, tissue, and urine samples obtained from cancer patients were compared with those obtained from healthy individuals, and lactic acid levels in gastric cancer patients were increased; differences in the metabolic activities of gastric precancerous lesions across different stages were found, which may be useful for screening (Yu et al., 2011; Abbassi-Ghadi et al., 2013). However, due to differences in specimen preparation and detection platforms, numerous restrictions on the use of metabolomics for the detection of biomarkers associated with gastric cancer remain.

The inhibition of the Warburg effect can suppress the growth of gastric cancer cells and reduce the level of drug resistance, thereby directly or indirectly exerting treatment effects. A ketogenic diet comprises high fats, low proteins, and low carbohydrates. A ketogenic diet can be used as adjuvant therapy for cancer as it increases the levels of cellular oxidative stress and lipid metabolism, thereby inhibiting glucose metabolism. Evidence suggests that a ketogenic diet can reduce tumor growth in mice with gastric cancer (Otto et al., 2008). Chemotherapy is the main treatment for advanced gastric cancer, and drug resistance results in low chemotherapeutic efficiency. With metabolomics, the metabolic patterns of anti-cancer drugs can be clarified, and the effectiveness of chemotherapy can be evaluated based on metabolic levels (Bayet-Robert et al., 2010). A previous study showed that 3-bromopyruvic acid (3-BrPA) and sodium citrate (SCT) significantly inhibit the proliferation of gastric cancer cells and glycolysis in orthotopic xenografts of gastric cancer (Wang et al., 2018). Citrate, a PFK inhibitor, showed strong inhibition of gastric cancer cell growth in BGC-823 and SGC-7901 lines and promoted the mitochondria apoptosis pathway (Lu et al., 2011). These compounds can be potentially used as therapeutic drugs for gastric cancer treatment but their specific mechanisms of action and the level of associated clinical risk need to be clarified. Many phytochemicals can kill gastric cancer cells *via* the inhibition of glycolysis; these are focused on in this review.

## 4 Methodology

We conducted an extensive literature search to identify relevant studies in a comprehensive manner. The researchers searched databases including the Web of Science, PubMed, Science Direct, Sci-Finder, SpringerLink, Embase, and EBSCO, and collated the literature for this review. The search was conducted until 30 June 2022, and at least two authors of this study addressed the search strategy to minimize the loss of relevant literature. Database-specific index terms (such as MeSH terms in PubMed) were combined with free-text words from the title or abstract for search terms. The target articles included the following three types of subject words and free words: 1) “Gastric cancer” and its synonyms, including “stomach neoplasm,” “stomach cancer,” and “gastric tumor.” 2) “Glycolysis” and its synonyms, including the “Embden-Meyerhof pathway” and “Warburg effect.” To expand the scope of the search process, we also included articles containing “HIF-1,” “HK2,” “LDH,” and “GLUT.” 3) “Phytochemical” and its synonyms, including “phytonutrient,” “herb,” “biological,” “plant-derived,” “natural product,” “medicinal plant,” and “plant bioactive compound.” During the screening process, non-experimental articles and articles that were not related to the topic were excluded. Some articles only reported on the effects of plant compounds on glycolysis-related enzymes or genes in gastric cancer cells and did not directly involve the measurement of changes in glycolytic flux in gastric cancer cells. We classified the compounds according to their molecular structure features. In each section, we rank the experiments from high to low according to their overall quality and relevance to the topics in this article.

## 5 Phytochemicals against glycolysis in gastric cancer cells

### 5.1 Phenols

Phenols are found in several plants, and the aromatic ring with at least one hydroxyl group is a structural feature of such compounds (Gan et al., 2019). Based on their chemical structures, phenolic compounds can be classified into different subgroups, including phenolic acids, flavonoids, tannins, coumarins, lignans, quinones, stilbenes, and curcuminoids (Tungmunnithum et al., 2018). Phenols can be classified as monohydric, dihydric, and polyhydric based on the number of hydroxyl groups attached to the aromatic ring. Phenols have a wide range of pharmacological effects, including anti-oxidant, anti-bacterial, anti-inflammatory, anti-cancer, and organ-protective properties (Tsao, 2010; Działo et al., 2016; Dei Cas and Ghidoni, 2018).

#### 5.1.1 Rosmarinic acid

Rosmarinic acid (RA) is widely found in plants, including Labiatae, *Arnebiaceae*, *Cucurbitaceae*, *Tiliaceae*, and *Umbelliferae*. It is a phenolic compound that was first extracted from *Rosmarinus officinalis*. RA has proven anti-inflammatory, anti-bacterial, anti-viral, anti-oxidant, and anti-tumor activities (Noor et al., 2022). RA inhibits MKN-45 gastric cancer cells activity in a dose-dependent manner with an IC50 of 240.2 μM. RA at 2 mg/kg/d also successfully inhibits tumor growth in MKN-45 tumor-bearing mice without reducing mice body weight. RA can inhibit the glycolytic activity in MKN-45 cells, reduce glucose consumption, and lactic acid production, and has also been confirmed in an *in vivo* experiment in tumor-bearing mice. RA simultaneously inhibits the expressions of HIF-1α, IL-6, and signal transducers and activators of transcription 3 (STAT3), along with the phosphorylation of STAT3, suggestive of glycolytic inhibition in gastric cancer cells through the IL-6/STAT3 pathway (Han et al., 2015).

#### 5.1.2 Curcumin

*Curcuma longa* is a *Zingiberaceae* plant species that grows in Asia. Its rhizome is used in traditional Chinese medicine (TCM). It is capable of treating pain, menstrual disorders, and other diseases. Curcumin is a polyphenolic compound found in various plants belonging to *Zingiberaceae* and *Araceae* families. Modern pharmacological studies have confirmed that curcumin has anti-oxidative, anti-inflammatory, anti-allergic, anti-depressive, and neuroprotective effects. Curcumin can inhibit various cancers, including those of the lungs, breasts, and colorectum (Patel et al., 2020; Ruiz de Porras et al., 2021; Lopresti, 2022). Curcumin significantly inhibits the growth (the IC50 is about 40 μg/ml), proliferation, and colony formation of gastric cancer cell lines, SGC-7901 and BGC-823, and significantly weakens tumor growth *in vivo*. After treatment with curcumin at 10 μg/ml, the extracellular acidification rates of gastric cancer cells reduce significantly, along with the base glycolysis rate. This may be related to rapid ROS production, leading to the phosphorylation of serine 15 of p53. Curcumin at 25 mg/kg/d effectively reduces the tumor weight of tumor-bearing mice without reducing mice body weight. (Wang L. et al., 2017). P53 can activate TIGAR, and TIGAR levels can reduce the expression of F-2,6-BP in cells. F-2,6-BP is the positive allosteric effector of PFK-1 (Bensaad et al., 2006). PFK-1 catalyzes the production of fructose-l, 6-bisphosphate (F-1,6-BP) from fructose-6-phosphate, a key rate-limiting enzyme in glycolysis. Fru-2,6-BP is also an inhibitor of fructose-1,6-bisphosphatase-1 (FBPase-1) that hydrolyzes F-1,6-BP to phosphate and fructose-6-phosphate (F6P), key enzymes in glycolysis. Increased expression of TIGAR leads to a decrease in the levels of F-2,6-BP in cells and a reduced cell glycolysis rate.

As the systemic bioavailability of curcumin is poor, the use of an adjuvant to improve its bioavailability has far-reaching prospects. Curcumin micelles prepared using amphiphilic Pluronic F-127 inhibit proliferation and promote apoptosis in gastric cancer cell lines, SGC-7901 and BGC-823, while simultaneously inducing ROS production and inhibiting mitochondrial respiration and aerobic glycolysis rates in gastric cancer cells. Curcumin micelle treatment significantly attenuates tumor growth *in vivo* in a nude mouse model (Lin et al., 2020). Another experiment compared the effects of curcumin and the monocarbonyl analog compound of curcumin, 1-(4-hydroxy-3-methoxy phenyl)-5-(2-nitrophenyl) penta-1,4-Dien-3-one (WZ35), on gastric cancer cell lines, BGC-823 and SGC7901. Curcumin can inhibit the proliferation of BGC-823 and SGC7901 cells and arrest the cell cycle in the G2/M phase. WZ35 showed stronger cell inhibition than curcumin, and significantly attenuated the proliferation of gastric cancer cells and induction of apoptosis, the growth suppression rate of WZ35 at 10 μg/ml to BGC-823 and SGC-7901 is about 80%. Curcumin and WZ35 at 25 mg/kg/d also inhibite the growth of gastric cancer in nude mice without reducing the weight of mice *in vivo*; however, the latter showed higher inhibition. WZ35 at 10 μg/ml also can reduce the extracellular acidification rate, inhibit cell glycolysis, and significantly reduce the expressions of cellular HK1 and G6PD proteins. WZ35 can increase the levels of ROS and c-Jun N-terminal kinase (JNK), while decrease the expression of Yes-associated protein (YAP) in patients with gastric cancer, indicating that WZ35 may inhibit glycolysis by activating ROS, resulting in the regulation of the YAP-JNK pathway (Chen T. et al., 2020).

#### 5.1.3 Catechin

Green tea is obtained from the leaves and buds of *Camellia sinensis* and is consumed worldwide. The numerous phenolic compounds isolated from green tea are collectively known as tea polyphenols, and these are the main active ingredients. Tea polyphenols exhibit anti-oxidative, anti-inflammatory, anti-bacterial, and anti-cancer effects and prevent cardiovascular diseases (Xing et al., 2019). Catechin, a tea polyphenol, can inhibit glycolysis in gastric cancer cells resistant to 5-Fluorouracil (5-FU) at 10 μM dose. The cell viability of drug-resistant cells was still greater than 50% when treated with 50 μM catechin. In comparison with the gastric cancer cell line, SNU620, 5-FU-induced SNU620/5-FU drug-resistant cells exhibit stronger glycolytic characteristics, higher levels of LDHA expression, higher extracellular acidification, and growth rates. During glycolysis, LDHA catalyzes the conversion of pyruvic acid to lactic acid, a key enzyme of the glycolytic pathway. Catechin treatment results in decreased LDHA activity in SNU620/5-FU cells, along with reduced production of lactic acid. The results of the molecular docking process suggest that catechins may interact with the T94, A95, Q99, R105, S136, R168, H192, and T247 residues of LDHA, thus interfering with its binding to the pyruvic acid substrate and inhibiting aerobic glycolysis (Han et al., 2021).

### 5.2 Flavonoids

Flavonoids are a class of secondary metabolites that are widely found in plants (Dixon and Pasinetti, 2010). Its basic carbon skeleton is composed of a C_6_-C_3_-C_6_ unit with two benzene rings (A and B) with a phenolic hydroxyl group connected through three carbon atoms. Based on the structural differences in their C_3_ atom, flavonoids can be divided into multiple subclasses. These exert a wide range of biological activities, including anti-oxidative, anti-bacterial, anti-inflammatory, and anti-cancer effects (Al-Khayri et al., 2022; Shamsudin et al., 2022; Shen et al., 2022).

#### 5.2.1 Wogonin

The rhizome of *Scutellaria baicalensis*, a perennial herb native to Asia, is used to treat inflammation in TCM. Modern pharmacology proves that the extract of *Scutellaria baicalensis* has neuroprotective, anti-bacterial, anti-inflammatory, antioxidant, and anti-tumor properties (Pei et al., 2022). Wogonin is the main component of *Scutellaria baicalensis*, and its *in vitro* effects on the gastric cancer cell line, SCG-7901, have been confirmed. Wogonin at 15 μg/ml reduces the expression of HIF-1α, and down-regulates the levels of MCT4 and LDH, thereby reducing the production of lactic acid, improving the acidic microenvironment, inhibiting cellular proliferation, and the IC50 was about 18.17 μg/ml (Wang et al., 2019).

#### 5.2.2 Baicalein

Baicalein, another major component of *Scutellaria baicalensis*, can inhibit glycolysis in gastric cancer cells through the PTEN/Akt/HIF-1α signaling pathway. Baicalein at the dose below 40 µM had no significant toxicity to AGS cells, and the AGS cell viability was still greater than 60% after 48 h of treatment with 60 µM Baicalein. Baicalein reduces the levels of HIF-1α in AGS gastric cancer cells in a dose-dependent manner (10, 20, and 40 µM); down-regulates the levels of HK2, LDHA, and PDK1; inhibits Akt phosphorylation under hypoxic conditions; promotes the expression of PTEN protein, and gradually restores glucose uptake and lactic acid production in hypoxic AGS cells to those observed under normoxic conditions (Chen et al., 2015).

#### 5.2.3 Licochalcone A

*Glycyrrhiza uralensis Fisch.* is a perennial herb, widely distributed in the low-altitude regions of Asia. Its rhizome is used for the treatment of pain and respiratory diseases in TCM. Licochalcone A (Lic A) is the main component of *Glycyrrhiza uralensis Fisch.*, and exerts anti-oxidative, anti-inflammatory, anti-bacterial, hepatoprotective, and anti-tumor effects (Wang et al., 2015; Li X. et al., 2019; Wang Z. F. et al., 2020; Tian et al., 2022). The IC50s of Lic A on MKN45 and SGC-7901 cells were 63.57 µM and 55.56 µM, and the 180 µM Lic A had no obvious toxicity on GES-1 cells. In gastric cancer cell lines, 60 µM Lic A reduced the expression of HK2, decreased cell glucose consumption and lactic acid production, inhibited cell proliferation, and promoted apoptosis. These processes are related to the inhibition of the cellular Akt signaling pathway. In an *in vivo* experiment, 10 mg/kg/d Lic A inhibited the growth of the cancer cell MKN45 xenograft model tumor, the expression of HK2 in tumor tissues was reduced significantly, but did not affect that weight of the mice (Wu et al., 2018). HK2 is the first key rate-limiting enzyme in glycolysis that can catalyze the conversion of glucose to G6P and also antagonizes mitochondria apoptosis pathway (Garcia et al., 2019).

#### 5.2.4 Helichrysetin

*Alpinia katsumadai Hayata* is a mountain ginger plant, and its seeds are used in Chinese medicine. Helichrysetin (HLE), a chalcone isolated from *Alpinia katsumadai Hayata*, has anti-tumor activity against lung and cervical cancers *in vivo* and *vitro* experiments (Ho et al., 2013; Wang Z. et al., 2021). HLE significantly inhibited the activity of gastric cancer cells. The IC50s for MGC-803, AGS, and SGC-7901 cells were 16.07, 28.02, and 31.8 µM, respectively. HLE at 20 µM inhibited metabolic reprogramming by inhibiting the activity of c-Myc. After treatment with HLE, the phosphorylation of PI3K, Akt, mTOR, and P70S6K was inhibited, and the levels of c-Myc, PDH kinase 1 (PDHK1), and LDHA decreased markedly in MGC803 gastric cancer cells. Cellular mitochondrial oxidative phosphorylation was enhanced, and the extent of glycolysis and compensatory glycolysis along with the levels of lactic acid production and its release was reduced. Similar results have been shown *in vivo* in mice, HLE at 3, 10 and 30 mg/kg/d inhibits tumor growth in MGC803 cell xenografts without an obvious effect on body weight in nude mice. A significant reduction in the levels of c-Myc, PDHK1, and LDHA as well as the phosphorylation of PI3K, AKT, mTOR (Wang P. et al., 2022), and p70S6K in tumor tissues, inhibiting c-Myc-mediated metabolic reprogramming have been reported. The inhibition of PDHK1 increases the activity of PDH and promotes the entry of pyruvic acid into the mitochondria participating in the TCA, thereby inhibiting aerobic glycolysis (Golias et al., 2019).

#### 5.2.5 Luteolin

*Lonicera japonica* is a vine, and its flower is used in TCM, as it is considered to possess heat-clearing and detoxifying effects. Its main active ingredient, luteolin, is found in numerous fruits, vegetables, and herbs. Pharmacological studies show that luteolin has anti-inflammatory, cardioprotective, neuroprotective, and anti-tumor effects. Luteolin can inhibit the proliferation of AGS, BGC-823, and SGC-7901 gastric cancer cells and cause cell cycle arrest in the G1 phase (Luo et al., 2017; Conti et al., 2021; Franza et al., 2021; Kempuraj et al., 2021). Luteolin inhibited the proliferation of gastric cancer cells (AGS, BGC-823, SGC-7901) in a dose-dependent manner, and its IC50s were about 20–25 μM. Luteolin at 5, 10 and 50 μM up-regulates miR-34a expression in gastric cancer cells, which in turn targets the down-regulation of HK1, significantly increases the cellular expressions of p53 and p21, and decreases the phosphorylation of MEK and ERK. Similar results have been shown *in vivo* in a BGC823 cell xenograft model, but the weight changes of mice were not recorded. The effect of luteolin on the glycolytic flux has not been directly observed and needs to be examined in future studies (Zhou et al., 2018).

#### 5.2.6 Total flavones of Selaginella uncinata

*Selaginella uncinata*, a fern found in Southern China, is used in TCM for the treatment of inflammation and trauma. Total flavonoids of *Selaginella uncinata* (TFS) represent one of the main active components of *Selaginella uncinata*, and have been proven to exhibit anti-inflammatory and anti-hypoxic effects (Zheng et al., 2011; Yu et al., 2017). In an *in vivo* experiment, gastric cancer AGS cells were treated with 5, 15, and 25 μg/ml TFS and found that TFS could inhibit the proliferation of AGS cells (the IC50 is about 15–25 μg/ml), promote apoptosis, and inhibit cell glycolysis. Studies focusing on the mechanisms underlying these phenomena have shown that TFS plays an anti-cancer role by down-regulating the expression of circ_0009910 (Zhang and Yu, 2020). Circ_0009910 is a target for the treatment of gastric cancer, and can promote the proliferation, metastasis, and glycolysis of gastric cancer through the miR361-3p/SNRPA axis (Liu et al., 2022).

### 5.3 Phenylpropanoids

Phenylpropanoids are the largest category of secondary metabolites in plants, containing one or more C_6_-C_3_ groups. Based on their structural characteristics, phenylpropanoids can be classified as simple phenylpropanoids, coumarins, lignans, *etc.* Broadly, flavonoids are also considered phenylpropanoid derivatives. Phenylpropanoids have several biological activities, including anti-oxidative, anti-inflammatory, and anti-cancer effects.

#### 5.3.1 Podofilox

Podofilox is a medicinal component found in various *Berberidaceae* plants, including *Podophyllum peltatum L.*, distributed across North America, and *Dysosma versipellis*, distributed across China. Multiple experiments suggest that podofilox and its derivatives have anti-viral and anti-cancer effects and can inhibit the growth of multiple cancer cells (Beutner et al., 1989; Cohen et al., 2016; Zhao et al., 2019). In gastric cancer cell lines, AGS and HGC-27, podofilox inhibits cell proliferation, IC50s were 3.409 and 3.349 nM, respectively. Podofilox at 3.4 nM also inhibited colony formation and reduces the levels of HK2, PKM2, c-Myc, and ATG10. Thus, podofilox inhibits the proliferation of gastric cancer cells by regulating the c-Myc/ATG10 signaling axis (An et al., 2021).

#### 5.3.2 Paeonol

*Paeonia suffruticosa Andr* is a deciduous shrub native to China and is currently cultivated worldwide. Paeonol is a major pharmaceutical ingredient isolated from the root bark of *Paeonia suffruticosa Andr* and has been used in various clinical preparations owing to its anti-inflammatory and pain relief-related properties (Zhang et al., 2019). Paeonol also has neuroprotective, anti-tumor, and anti-cardiovascular effects. Paeonol inhibited activity of Apatinib (AP)-resistant gastric cancer cells in a dose-dependent manner with an IC50 of 63.64 mg/L for BGC-823/AP and 56.83 mg/L for MGC-803/AP. Paeonol can also inhibit the migration, invasion, and glycolysis in lapatinib-resistant BGC-823/AP and MGC-803/AP gastric cancer cells. After treatment with paeonol at 60 mg/L, the levels of GLUT1, LDHB, and HK2 proteins in gastric cancer cells decrease markedly, and aerobic glycolysis is significantly weakened. Paeonol can up-regulate miR-665 levels by inhibiting the expression of LINC00665, and further attenuating the expression of MAPK1. In gastric cancer, LINC00665, miR-665, and MAPK1 form a ceRNA network that promotes the development of resistance in gastric cancer cells. Paeonol down-regulates the levels of GLUT1, LDHB, and HK2 by regulating the LINC00665/miR-665/MAPK1 axis, which in turn inhibits glycolysis. *In vivo* experiments confirm the growth inhibitory effects of paeonol at 50 mg/kg/d on the lung metastatic nodules in BGC-823/AP cell transplanted nude mice (Li et al., 2022).

#### 5.3.3 Salidroside

*Rhodiola Rosea* is widely distributed at high altitudes, and its rhizome can invigorate qi and blood according to TCM. Salidroside is the main active component of *Rhodiola rosea*. Studies have shown that salidroside has anti-inflammatory, anti-oxidative, anti-hypoxic, anti-cancer, immune-stimulatory, cardioprotective, and brain protective properties effects (Zhang et al., 2021). Following treatment with salidroside at 80 μM (at this dose, the cell viability is more than 60%), the levels of PKM2, GLUT1, and enolase 1 (ENO1) in SGC-7901 and MKN-45 gastric cancer cells decrease markedly, and glycolysis is inhibited. Salidroside at 50 mg/kg/d significantly inhibits tumor growth, reduced the ENO1, HK2, and GLUT1 protein expression levels in MKN-45 and SGC-7901 cell xenografted mice *in vivo* (Dai et al., 2021). ENO1, a key enzyme in the glycolytic pathway, catalyzes the interconversion of 2-phospho-D-glycerate and phosphoenolpyruvate. ENO1 is a multifunctional protein with carcinogenic properties that promotes cell proliferation, migration, and invasion (Huang et al., 2022).

#### 5.3.4 β-asarone

*Acorus calamus* is used in many ethnic medicinal systems to treat nervous system diseases, inflammation, and digestive system disorders. β-asarone is mainly found in the rhizome of *Acorus calamus L.* and has several pharmacological activities, including anti-inflammatory, anti-oxidative, central nervous system inhibiting, anti-diarrheal, anti-bacterial, and anti-cancer effects (Rajput et al., 2014). β-asarone significantly inhibits cell activity, IC50s in MGC803, SGC7901, and MKN74 cells are 39.92, 84.6, and 96.22 μg/ml, respectively. β-asarone can significantly inhibit the activity of LDH in SGC-7901 and MKN-74 gastric cancer cells, induce apoptosis in a hypoxic environment, and arrest cellular proliferation in the G2/M phase. β-asarone inhibits STAT5 phosphorylation and the expressions of HIF1-α, c-Myc, and p-PDK1 in MGC803 and SGC7901 cells, without resulting in any significant changes in the PDK1 levels (Tao et al., 2020). PDK1 can prevent pyruvate from being converted into acetyl-CoA by inhibiting the activity of LDH and preventing energy from entering the TCA and resulting in oxidative phosphorylation. β-asarone inhibits glycolysis by down-regulating the STAT5/c-Myc signaling pathway and inhibiting the phosphorylation of PDK1 in combination with HIF-1α signaling (Dang, 2007; Wang N. et al., 2022).

### 5.4 Terpenoids

Terpenoids are a large class of isoprenoid derivatives that exist widely in nature and are mainly isolated from plants, microorganisms, and marine organisms. The general formula of terpenes is (C_5_H_8_)_n_ (where n is the number of units of isoprene) (McGarvey and Croteau, 1995; Lange and Ahkami, 2013). More than 50,000 terpenes have been found, which have a wide range of biological functions, including anti-inflammatory, anti-allergic, and preventative disease properties, and are used for the treatment of cardiovascular and cerebrovascular diseases (Singh and Sharma, 2015). Terpenes exert extensive anti-cancer effects. Paclitaxel, the anti-cancer drug commonly used in the clinic, is a tricyclic diterpenoid extracted from the *Taxus* plants (Jennewein and Croteau, 2001).

#### 5.4.1 Crocin

*Crocus sativus L.* is mainly distributed across Europe, North Africa, and West Asia. The stigma from its flower can be used as a spice. It promotes blood circulation and is used for treating depression in TCM (Ahrazem et al., 2015). Crocin, the main active ingredient of *Crocus sativus L*, has various pharmacological effects, including anti-bacterial, anti-oxidative, anti-depressive, and cardioprotective properties; it lowers blood lipid levels (Butnariu et al., 2022). Crocin at 2–3 μM reduces the expression of HIF-1α in AGS and HGC-27 gastric cancer cells by inhibiting the miR-320/Krüppel-like factor 5 (KLF5)/HIF-1α signaling axis. miR-320 is an important anti-tumor factor in gastric cancer. KLF5, a member of the KLF family of transcription factors, can regulate several important target genes, including HIF-1α. KLF5 is a target gene of miR-320, as evidenced by bioinformatic analysis and dual-luciferase reporter assay. Crocin up-regulates the levels of miR-320, reduces the expression of KLF5, and reduces the levels of HIF-1α, thereby inhibiting the migration, invasion, and epithelial-mesenchymal transition (EMT) of gastric cancer cells (Zhou et al., 2019). The results of an enzymatic assay confirmed the inhibition of LDH by crocin. Cronin, a derivative of crocin, shows high inhibition of LDH (Granchi et al., 2017).

#### 5.4.2 Oleanolic acid

OA is a triterpenoid found in *Oleaceae* plants. It is mainly extracted from the leaves of *Olea europaea L.* and the fruit of *Ligustrum lucidum ait*. Modern pharmacological studies have proven the anti-bacterial, anti-inflammatory, and anti-cancer effects of OA (Castellano et al., 2022). OA at 30 μM has no toxicity to GES-1 cells, while IC50 of OA is about 20–30 μM in MKN-45 and SGC-7901 cells. OA at 10, 20, and 30 μM reduces the levels of glycolysis-related activities, including cell glucose uptake and consumption, intracellular lactic acid levels, and extracellular acidification rate, in MKN-45 and SGC-7901 gastric cancer cells. Simultaneously, OA down-regulates the expression of HIF-1α in cells, reduces the nuclear abundance of HIF-1α and YAP, and reduces the expression and activity of the glycolysis rate-limiting enzymes, HK2 and PFK1. YAP can control the expression of HIF-1α in gastric cancer cells at the transcriptional level. The up-regulation of HIF-1α further increases the expression of the glycolysis-related rate-limiting enzymes. OA blocks HIF-1α-mediated glycolysis in gastric cancer cells by inhibiting YAP (Li Y. et al., 2019).

#### 5.4.3 Tanshinone IIA

*Salvia miltiorrhiza* is a traditional herb used widely in Asia. It promotes blood circulation and relieves pain according to TCM. The main active ingredient of *Salvia miltiorrhiza* is an abietane-type diterpene with antioxidative, anti-inflammatory, and anti-tumor activities (Ryu et al., 1997). Tanshinone IIA (TIIA) is a diterpenoid quinone extracted from the root of *Salvia miltiorrhiza*. The IC50 of TIIA in gastric cancer AGS cells was 5.3 μM. An integrated transcriptomic and proteomic analysis revealed that TIIA at 5.3 μM reduced glucose consumption and the production of ATP and pyruvic acid by down-regulating the expression of glucose-6-phosphate isomerase (G6PI) and LDHB in AGS gastric cancer cells. TIIA also led to increased p53 and decreased Akt levels, cell cycle arrest, apoptosis, and inhibition of proliferation. G6PI catalyzes the reversible isomerization of G6P to F6P. The down-regulation of G6PI can reduce cell glucose consumption, thus resulting in the inhibition of glycolysis. LDHB is a subunit of LDH. The down-regulation of LDHB inhibits the conversion of lactic acid to pyruvic acid, which in turn inhibits gluconeogenesis and the TCA cycle and reduces the synthesis of acetyl-CoA, lipids, and non-essential amino acids (Lin et al., 2015b). The furan ring is a structure specific to tanshinone (Wang and Peters, 2022). An experiment was conducted to compare numerous fir-type diterpenoids extracted from the root of *Salvia miltiorrhiza*, including Sibiriquinone A, Sibiriquinone B, Cryptotansinon, and Dihydratansinone I which could reduce the levels of HIF-1α in hypoxia-induced AGS cells. The inhibition of HIF-1 was more significant when the furan ring was saturated, and the substitutions and quinone conjugations in the abietane skeleton influenced the inhibitory activities of the compounds (Dat et al., 2007).

#### 5.4.4 Cardiac glycosides

CGs, including ouabain, oleandrin, and digoxin, are a class of glycosides with cardiotonic effects that are often used for the treatment of heart failure and arrhythmia. Such compounds can be extracted from plants. Ouabain is usually obtained from *Strophanthus gratus*; oleandrin is the active ingredient in *Nerium oleander L.*, and digoxin is the main active ingredient in *Digitalis purpurea* and *Digitalis lanata* (Whayne, 2018). Accumulating evidence shows that CGs have significant anti-cancer effects (Shah et al., 2022). *In vitro* experiments have confirmed that CGs reduce the levels of GLUT1 in MKN45 cells, and induce dynamin-dependent GLUT1 endocytosis, resulting in subsequent GLUT1 degradation in lysosomes, which significantly affects the uptake of glucose by cancer cells, thereby inhibiting glycolysis in gastric cancer cells (Fujii et al., 2022).

#### 5.4.5 DT-13

*Ophiopogon japonicus* is a perennial herb distributed across Asia and has a long medicinal history. DT-13, an active component of *Ophiopogon japonicus*, has anti-inflammatory, immune regulatory, and anti-cancer properties, and protective effects on blood vessels (Chen et al., 2016). *In vitro* experimental findings confirm that DT-13 at 10 μM combined with Topotecan at 0.1 or 1 μM significantly inhibits tumor growth and glycolysis in BGC-823 cells, and promotes tumor cell apoptosis. This has also been proved *in vivo* in BGC-823 xenograft mice with the dosage of DT-13 at 1.25 mg/kg/d combined Topotecan at 0.5 mg/kg/d. Mechanistic studies show that combination treatments promote the degradation of epidermal growth factor receptors through the activity of non-muscle myosin IIA, thus inhibiting HK2 activity, simultaneously suppressing the specific binding of HK2 to mitochondria, and attenuating aerobic glycolysis (Yu et al., 2019).

#### 5.4.6 Celastrol

The roots, leaves, and flowers of *Tripterygium wilfordii*, a climbing shrub plant distributed in East Asia, are used as herbal medicines in TCM. Celastrol is an active ingredient in *Tripterygium wilfordii* that has cardioprotective, neuroprotective, anti-inflammatory, and anti-cancer effects (Hou et al., 2020). *In vitro* experiments confirmed that celastrol could inhibit the proliferation of BGC-823 cells, the IC50s at 24, 48, and 72 h were 1.66, 1.63, and 1.32 μM, respectively. The follow-up experiment was treated with 0.72/1.0/1.5 μM for 48 h. Celastrol at 0.72, 1.0, and 1.5 μM can also promote apoptosis, and inhibit glucose consumption and lactic acid production. Studies on the mechanisms underlying these phenomena have shown that celastrol inhibits the expression of GLUT1, HK2, and LDH, and reduces the activity of HK and LDH, thereby inhibiting glycolysis in gastric cancer cells (Li K. et al., 2019).

#### 5.4.7 Kirenol

*Siegesbeckia orientalis L.* is a composite plant, widely distributed in Asia, Europe, and North America. Kirenol, a diterpenoid compound isolated from *Siegesbeckia orientalis L.*, has anti-inflammatory, immunosuppressive, anti-allergic, and anti-tumor activities (Wang Q. et al., 2021). *In vivo* experiments confirmed that kirenol at 30 mg/kg/d could effectively reduce the tumor volume and incidence of MNNG-stimulated gastric cancer in rats, reduce the levels of oxidative stress and inflammatory response through the NF-κB signaling cascade, and inhibit the expression of LDH in gastric cancer tissues, without reducing mice body weight. The effect of kirenol on the glycolysis rate in mouse tissue was not assessed in this study, and needs to be assessed in future studies (Liu W. et al., 2020).

### 5.5 Other phytochemicals

#### 5.5.1 Taraxasterol

*Taraxacum mongolicum* is a perennial herb with a wide distribution in the low-to-middle altitudes in Asia. In TCM, the entire herb is considered to clear heat and achieve detoxification; it is thus used to treat various inflammation-associated conditions. Taraxasterol is a plant sterol extracted from the root of *Taraxacum mongolicum* (Sharifi-Rad et al., 2018; Yang F. et al., 2021). It shows anti-bacterial, anti-inflammatory, and anti-tumor properties. Taraxasterol with 0–20 μM can inhibit proliferation (the IC50 is about 15 μM), glucose uptake, lactic acid production, and ATP levels, along with enhancing apoptosis in the gastric cancer cell line, HGC-27. Taraxasterol have no effect on the viability of GES-1 cells at the same dose. Taraxasterol can inhibit the mRNA and protein expressions of glycerol-3-phosphate dehydrogenase 2 (GPD2), and the expression of glycolysis-related enzymes, including HK2, LDHA, and PFKM, thereby inhibiting aerobic glycolysis (Zhao et al., 2021). GPD2 is a mitochondrial gene encoding mitochondrial glycerophosphate dehydrogenase (mGPDH). mGDPH, a glycerol-3-phosphate dehydrogenase, can catalyze the oxidation of glycerol-3-phosphate (G3P) to dihydroxyacetone phosphate (DAP). Its main metabolic activity is the re-oxidation of the cytosolic nicotinamide adenine dinucleotide (NADH) produced by glycolysis. This allows for sustained production of cytosolic ATP without the accumulation of intermediate byproducts, including lactic acid (Mracek et al., 2013).

## 6 Critical considerations

### 6.1 Toxic side effects of phytochemicals

Although several studies have verified the inhibitory effects of phytochemicals *via* the glycolytic pathway on the growth of gastric cancer cells, a major concern is elucidating the biological toxicity of phytochemicals along with their therapeutic effects. A dose of 147.75 μM or higher of glycyrrhizin chalcone A is cytotoxic to Chinese hamster ovary fibroblasts (de Freitas et al., 2020). High doses (200–300 mg/kg) of sulforaphane can cause dyskinesia, decreased skeletal muscle strength, leukopenia, or even death in mice (Socala et al., 2017). Intraperitoneal administration of wogonin (100 mg/kg) aggravated DSS-induced colitis in mice (Xiao et al., 2017). The teratogenic concentration of TIIA on zebrafish embryos is nearly 1 μM (Wang T. et al., 2017). β-asarone exhibits notable organ toxicity, reproductive toxicity, and carcinogenicity (Benny et al., 2017; Uebel et al., 2021). Existing cellular experiments have been conducted *in vitro*, and *in vivo* data are necessitated to study the toxicology and pharmacokinetics of phytochemicals, to determine safe and reasonable therapeutic doses and administration methods.

The inhibition of glycolysis may suppress bodily functions. Glycolysis is the main mode by which the body obtains energy when it is relatively deprived of oxygen and is the main source of energy for muscles during strenuous exercises (De Feo et al., 2003). Inhibition of glycolysis can result in decreased exercise tolerance in muscles. Glycolysis is the main mode through which nerve cells (Tang, 2020), white blood cells (Madden and Rathmell, 2021), and bone marrow cells (van Noorden et al., 2022) obtain energy upon the availability of adequate oxygen, and it is also the only means through which mature red blood cells obtain energy. The inhibition of glycolysis might affect the regular functioning of these cells. Glycolysis is the pathway responsible for the degradation of fructose, galactose, and other hexoses (Baker and Winegrad, 1970). The inhibition of glycolytic processes alone might inhibit regular metabolic functions and cause damage to the body.

Drugs targeted against glycolysis must be used in combination with specific treatments to ensure that these work against tumor cells only. Nano drugs can facilitate targeted treatment at specific sites in the body. The doxorubicin (Dox)-based liposome nanosystem constructed by Yang et al. (2020) was used to administer tumor-specific chemotherapy *via* different stress sensitization techniques in cancerous and normal cells. Ding et al. (2021) also developed a ‘‘Sweet Tooth”-oriented SN38 prodrug delivery nanoplatform (Glu-SNP) for gastric cancer treatment based on the specific targeting of cancer cells exhibiting GLUT1 expression.

### 6.2 Bioavailability and herbal nanomedicines

Poor oral bioavailability is a problem associated with several plant-derived compounds. A phase I clinical trial assessed the bioavailability of curcumin and found that the peak concentrations of curcumin in the patient sera were 0.51 ± 0.11 μM, 0.63 ± 0.06 μM, and 1.77 ± 1.87 μM after administration of daily doses of 4,000 mg, 6,000 mg, and 8,000 mg curcumin, respectively (Cheng et al., 2001). Herbal nanomedicines (HNMs) refer to pharmaceutical particles with a diameter of 10–200 nm. These are produced by combining herbal ingredients with nanoparticles *via* techniques of nanotechnology. Nanoparticles have unique chemical, electrical, structural, magnetic, mechanical, and biological properties that increase the solubility of poorly soluble drugs and enhance tissue/membrane permeability. These nanoparticles can be used for the delivery of herbal drugs to target tissues in a precise and controlled manner. At present, HNMs including metal nanoparticles, polymer nanoparticles, phytic acid bodies, liposomes, alcohol-soluble bodies, nanoemulsions, micelles, and dendrimers have been employed (Teja et al., 2022). For example, the use of polyamidoamine dendrimers improves the pharmacokinetic parameters of berberine whilst reducing drug toxicity (Gupta et al., 2017).

### 6.3 Clinical transformation and application

Several challenges are associated with the translation of pre-clinical findings to derive clinical implications. First, the specific mechanisms of action of some phytochemicals need verification. The occurrence and development of gastric cancer are complex, and involve multiple molecules; aerobic glycolysis is also regulated by several molecules. Further studies are needed to examine the specific target molecules of these drugs, the affected underlying pathways, and multi-pathway interactions. It is necessary to understand the structure-activity relationship of compounds based on their molecular structures. This will aid the identification of effective compounds and guide the development of new drugs. The methods used for extracting chemicals from plants need improvement. Commonly used extraction methods for plant compounds include impregnation, distillation, Soxhlet extraction, ultrasonic-assisted extraction, and microwave-assisted extraction. The selection of the appropriate extraction method can aid the extraction of the stable specified compounds and obtain many such compounds for research purposes (Idris and Mohd Nadzir, 2021; Chang et al., 2022). The protection of plant resources is a problem that requires considerable attention. *Rhodiola sachalinensis* and many medicinal plants have been listed as vulnerable species in China due to their excessive commercial exploitation (Brinckmann et al., 2021). Examining chemical synthesis-based alternatives to plant extraction is important, as these can stably produce the required compounds and help protect the plant population.

Many natural compounds were found to regulate glycolysis for gastric cancer therapy, some of them are currently in different stages of preclinical and clinical trials. Curcumin is a multi-functional molecule. Many clinical studies on inflammation, tumor, cardiovascular and other diseases have confirmed its safety and effectiveness, and the maximum intake dose of curcumin can be up to 12 g/d (Jurenka, 2009; Gupta et al., 2013; Salehi et al., 2019). Crocin also has great potential for drug development, and shows a safe efficacy in the treatment of type II diabetes (Sepahi et al., 2022), anxiety and depression during chemotherapy (Chang and Teng, 2018), and hyperlipidemia (Roshanravan et al., 2022), with the generally used dose of 30 mg/d. Tea polyphenols such as catechin also shows exciting clinical potential. Catechin up to 1315 mg/d is considered safe, but it may have harmful effects in the inflammation of esophagus and colon (Yang and Zhang, 2019). Baicalein at 600 mg/d was confirmed to be serious adverse events-free, well tolerated, and characterized by pharmacokinetics in a randomized, double-blind trial (Li et al., 2021). OA has extensive anti-cancer activity, but its oral utilization is low, with the Cmax only 12.12 ± 6.84 ng/ml after oral administration of 40 mg of OA. The nano-drugs and drug analogues developed around OA may solve this problem (Shanmugam et al., 2014). RA at 500 mg/d was safe and well tolerated (Noguchi-Shinohara et al., 2015), and 10 mg/d β-asarone combined with other drugs was helpful for the treatment of Alzheimer’s disease without significant adverse reactions (Chang and Teng, 2018). A preparation of TIIA, Sodium Tanshinone IIa Sulfonate Group, has been used for the treatment of chronic cardiovascular diseases and a dose of 80 mg/d proved to be safe and effective (Mao et al., 2021).

## 7 Conclusion

Gastric cancer seriously affects human health and life, and the lack of effective drugs for targeted treatment is the main cause of its high mortality rate. Gastric cancer cells undergo aerobic glycolysis to rapidly obtain energy, produce synthetic raw materials for the generation of lipids and amino acids, and promote angiogenesis, thus achieving rapid proliferation. Targeted glycolysis is a promising approach for the treatment of gastric cancer. Herbal medicines have long been used in the treatment of diseases. About one-third of the developed drugs are derived from plants (Caesar and Cech, 2019), and the use of plant-derived compounds that target glycolysis is a feasible option for the treatment of gastric cancer.

**FIGURE 2:**
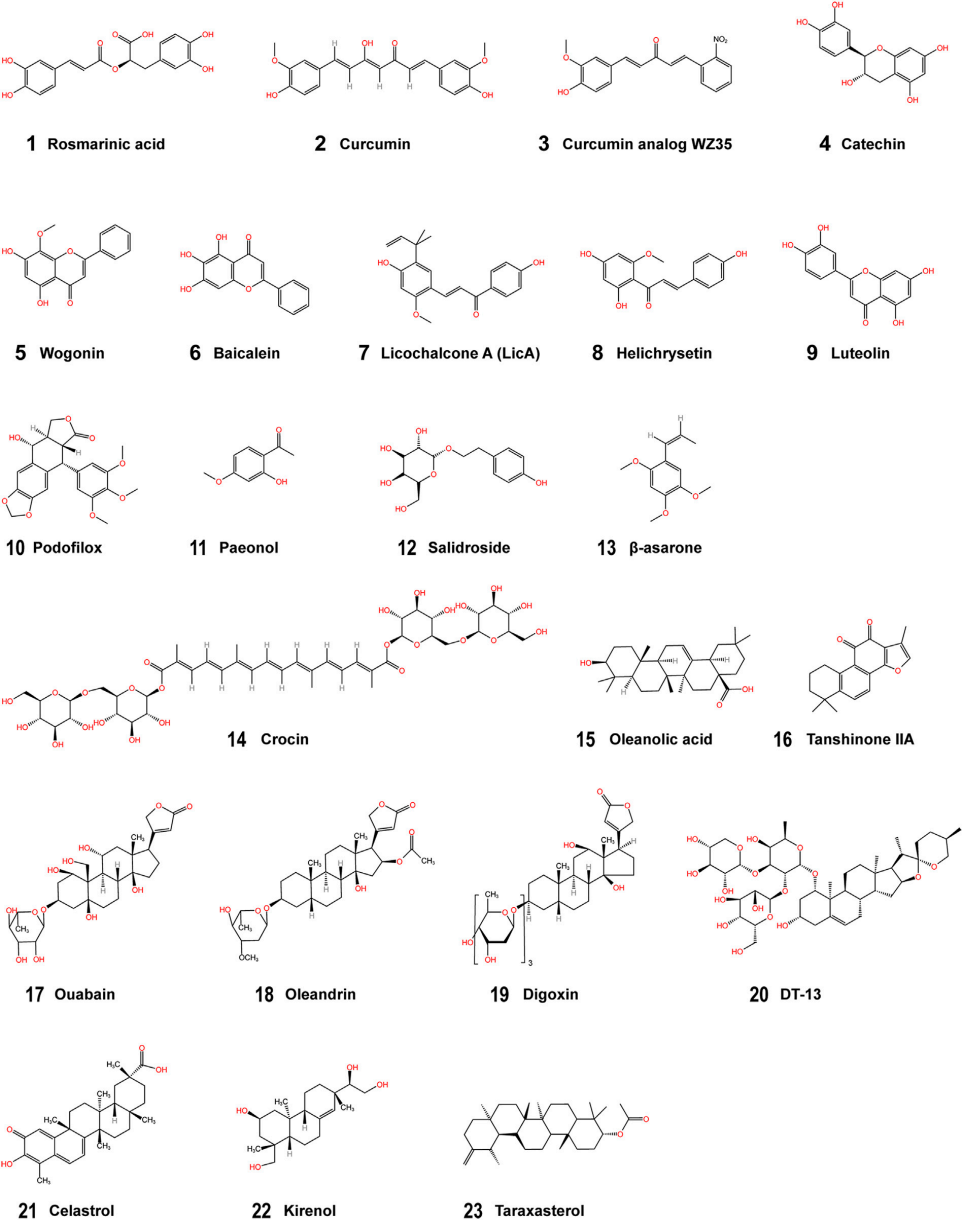
Structural formulae of the phytochemicals. **(1–4)** Phenolic compounds; **(5–9)** Flavonoids; **(10–13)** Phenylpropanoids; **(14–22)** Terpenoids; **(23)** Other phytochemicals.

**FIGURE 3:**
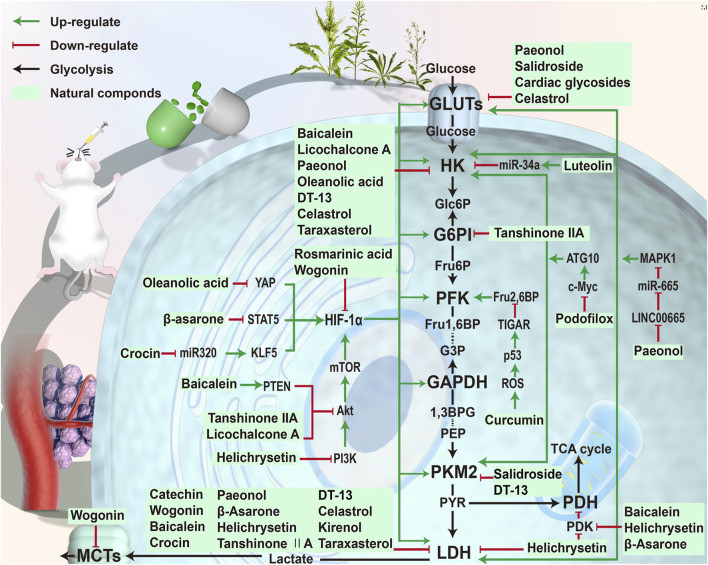
Phytochemicals targeting the glycolysis in gastric cancer. Phytochemicals were extracted from plants and tested *in vivo or in vitro*. These compounds affected the glycolysis process in gastric cancer cells through different ways. Baicalein and other compounds could directly regulate glycolysis-related enzymes. Compounds such as wogonin inhibited glycolysis by suppressing HIF-1α. Compounds such as curcumin regulated the glycolysis through other pathways.

In this review, we have identified some natural components that can inhibit glycolysis in gastric cancer cells and thus promote their apoptosis (Figures 2, 3, and Table 1). We structurally classified these phytochemicals and summarized the specific mechanisms by which they inhibit aerobic glycolysis in gastric cancer. HIF-1α is a key molecule that enhances the Warburg effect and is also the main target molecule for many natural products. Key proteins and enzymes in the glycolytic pathway, including GLUT, HK, PFK, and LDH also represent major targets for natural products that inhibit glycolysis in gastric cancer cells. Among many phytochemicals, terpenoids exhibited the most diverse anti-glycolysis properties. Oleanolic acid inhibited gastric cancer glycolysis by down-regulating the expression of HIF-1α, and celastrol inhibited the expression of GLUT1, HK2, and LDH, thereby inhibiting glycolysis in gastric cancer cells. Among the phenols, curcumin regulated the respiration of gastric cancer cells through rapid ROS production. Among the flavonoids, helichrysetin inhibited metabolic reprogramming by inhibiting the activity of c-Myc. Among phenylpropanoids, salidroside can inhibit the activities of multiple enzymes PKM2, GLUT1, and ENO1 in gastric cancer cells. These natural products had a prominent effect on the intervention of glycolysis and had high research value.

**TABLE 1:** Phytochemicals targeting the glycolysis for gastric cancer therapy. (↑ increase, ↓ decrease).

Compound	Source	Experimental model		Efficacy	Mechanism	References
		*In Vitro*	*In Vivo*			
Rosmarinic acid	*Rosmarinus officinalis*	MKN-45	MKN45 xenograft mice	Inhibition of proliferation and glycolysis	↓IL6/STAT3; ↓miR-155; ↓HIF-1α	Han et al. (2015)
Curcumin	*Zingiberaceae* and *Araceae*	SGC-7901; BGC-823	BGC xenograft mice	Inhibition of proliferation and glycolysis; Induction of apoptosis	↑ROS; ↑p53	Wang L et al. (2017)
Catechin	*Camellia sinensis*	SNU620	—	Induction of apoptosis; Inhibition of glycolysis	↓LDHA	Han et al. (2021)
Wogonin	*Scutellaria baicalensis*	SGC-7901	—	Inhibition of proliferation and glycolysis	↓HIF-1α; ↓MCT4; ↓LDH	Wang et al. (2019)
Baicalein	*Scutellaria baicalensis*	AGS	—	Inhibition of proliferation and glycolysis	↑PTEN; ↓Akt; ↓HIF-1α; ↓HK2; ↓LDHA; ↓PDK1	Chen et al. (2015)
Licochalcone A	*Glycyrrhiza uralensis Fisch*	MKN-45; SGC-7901	MKN-45 xenograft mice	Inhibition of proliferation and glycolysis; Induction of apoptosis	↓Akt; ↓HK2	Wu et al. (2018)
Helichrysetin	*Alpinia katsumadai Hayata*	MGC803; SGC7901	MGC-823 xenograft mice	Induction of apoptosis; Inhibition of glycolysis	↓c-Myc; ↓PI3K/Akt/mTOR/p70S6k; ↓PDHK1; ↓LDHA	Wang P et al. (2022)
Luteolin	*Lonicera japonica*	AGS; BGC-823; SGC-7901	BGC-823 xenograft mice	Inhibition of proliferation	↑miR-34a; ↓HK1; ↑p53/p21; ↓MEK/ERK	Zhou et al. (2018)
Total flavones of *Selaginella uncinata*	*Selaginella uncinata*	AGS	—	Inhibition of proliferation and glycolysis; Induction of apoptosis	↓circ_0009910	Zhang and Yu, (2020)
Podofilox	*Podophyllum peltatum L*	AGS; HGC-27	—	Inhibition of proliferation	↓c-Myc/ATG10; ↑p53; ↓HK2; ↓PKM2	An et al. (2021)
Paeonol	*Paeonia suffruticosa Andr*	BGC-823; MGC-803	BGC-823/AP xenograft mice	Inhibition of proliferation, invasion and glycolysis; Induction of apoptosis	↓LINC00665/miR-665/MAPK1	Li et al. (2022)
Salidroside	*Rhodiola Rosea*	SGC-7901; MKN-45	MKN-45 and SGC-7901 cell xenografts mice	Inhibition of proliferation, invasion and glycolysis; Induction of apoptosis	↓PKM2; ↓ENO1; ↓GLUT1	Dai et al. (2021)
β-Asarone	*Acorus calamus L*	MGC-803; SGC-7901; MKN74	—	Inhibition of proliferation; Induction of apoptosis	↓STAT5/c-Myc; ↓HIF-1α; ↓PDK1/4; ↓LDH	Tao et al. (2020)
Crocin	*Crocus sativus L*	AGS; HGC-27	—	Inhibition of invasion	↑miR-320; ↓KLF5; ↓HIF-1α↓LDH	Zhou et al. (2019), Granchi et al. (2017)
Oleanolic acid	*Olea europaea L*	MKN-45; SGC-7901	—	Inhibition of proliferation and glycolysis	↓YAP; ↓HIF-1α; ↓HK2; ↓PFK1	Li Y et al. (2019)
Tanshinone IIA	*Salvia miltiorrhiza*	AGS	—	Inhibition of proliferation and glycolysis	↑p53; ↓Akt; ↓G6PI; ↓LDHB	Lin et al. (2015b)
Ouabain Oleandrin Digoxin	*Strophanthus gratus Nerium oleander L. Digitalis purpurea*	MKN	—	Inhibition of proliferation and glycolysis	↓GLUT1	Fujii et al. (2022)
DT-13	*Ophiopogon japonicus*	BGC-823	BGC-823 xenografts mice	Inhibition of proliferation and glycolysis; Induction of apoptosis	↓NM IIA; ↓EGFR↓HK2; ↓PKM2; ↓LDHA	Yu et al. (2019)
Celastrol	*Tripterygium wilfordii*	BGC-823	—	Inhibition of proliferation and glycolysis; Induction of apoptosis	↓GLUT1; ↓HK2; ↓LDH	Li K et al. (2019)
Kirenol	*Siegesbeckia orientalis L*	—	MNG-stimulated GC in rats	Inhibition of tumor growth	↓NF-Κb; ↓LDH	Liu W et al. (2020)
Taraxasterol	*Taraxacum mongolicum*	HGC-27	—	Inhibition of proliferation and glycolysis; Induction of apoptosis	↓GPD2; ↓HK2; ↓LDHA; ↓PFKM	Zhao et al. (2021)

Several plant-derived compounds can inhibit glycolysis in other cancer cells, and whether these can also act on gastric cancer cells warrants further investigation. Some natural compounds, such as curcumin, crocin, catechin, *etc.*, have shown effects in preclinical or clinical studies, and their therapeutic potential in the field of gastric cancer deserves further exploration. The drug toxicology, pharmacokinetics, compound structure-activity relationships, drug-drug synergy, and nano-drug delivery systems for plant-derived compounds need to be studied before these can be used in clinical settings. The development of drugs with targeted and effective action and those that exhibit lower toxicity in the treatment of gastric cancer is necessitated in the future to improve the quality of life and survival rate of patients with gastric cancer.

## Glossary

Hp: *Helicobacter pylori*
MCT (s): monocarboxylate transporter (s)
GLUT (s): Glucose transporter (s)
HK (1/2): Hexokinase (1/2)
G6P: glucose 6-phosphate
PFK (-1): Phosphofructokinase (-1)
PK (M2): pyruvate kinase (M2)
LDH (A/B): lactate dehydrogenase (A/B)
PDH: pyruvate dehydrogenase
TCA: tricarboxylic acid
MMP (s): matrix metalloproteinase (s)
HIF-1 (α/β): Hypoxia-inducible factor-1 (α/β subunits)
PHs: prolyl hydroxylases
PI3K: phosphatidylinositol-4,5-bisphosphate 3-kinase
MAPK: mitogen-activated protein kinase
F-2, 6-BP: fructose-2,6-bisphosphate
TIGAR: TP53-induced glycolysis and apoptosis regulator
PDK (1/2): pyruvate dehydrogenase kinase (isozyme 1/2)
PPP: pentose phosphate pathway
G6PD: glucose-6-phosphate dehydrogenase
AMPK: adenosine monophosphate-activated protein kinase
PI3K: phosphoinositide 3-kinases
mTOR: mammalian targets of the rapamycin
ROS: reactive oxygen species
RA: Rosmarinic acid
STAT: signal transducers and activators of transcription
TCM: traditional Chinese medicine
F-1,6-BP: fructose-l,6-bisphosphate
FBPase-1: fructose -1,6- bisphosphatase-1
F6P: fructose-6-phosphate
WZ35: 1-(4-hydroxy-3-methoxy phenyl)-5-(2-nitrophenyl) penta-1,4-Dien-3-one
YAP: Yes-associated protein
JNK: c-Jun N-terminal kinase
EGCG: epicatechin-3-gallate
VEGF: vascular endothelial growth factor
5-FU: 5-Fluorouracil
EZH2: enhancer of zeste homolog 2
Lic A: Licochalcone A
TFS: Total flavonoids of *selaginella uncinat*
HLE: Helichrysetin
PDHK1: pyruvate dehydrogenase kinase 1
MAPK (1): mitogen-activated protein kinase (1)
ENO1: enolase 1
PGA: 2-phospho-D-glycerate
KLF5: Krüppel-like factor 5
EMT: Epithelial-Mesenchymal Transition
OA: Oleanolic acid
TIIA: Tanshinone IIA
G6PI: glucose-6-phosphate isomerase
GPD2: Glycerol-3-phosphate dehydrogenase 2
mGPDH: mitochondrial glycerophosphate dehydrogenase
G3P: glycerol-3-phosphate
DAP: dihydroxyacetone phosphate
NADH: nicotinamide adenine dinucleotide
